# Supplementary material for: Cortical encoding of melodic expectations in human temporal cortex
Source: eLife. 2020 Mar 3;9:e51784. doi: 10.7554/eLife.51784 (PMC7053998; doi:10.7554/eLife.51784)
Supplement: Supplementary file 1. [file elife-51784-supp1.docx]

| Electrode | Name | x (mm) | y (mm) | z (mm) |
| --- | --- | --- | --- | --- |
| e1 | Planum temporale | -64.641 | -16.029 | 3.617 |
| e2 | Planum temporale | -63.843 | -16.692 | 4.385 |
| e3 | Planum temporale | -63.088 | -17.038 | 4.779 |
| e4 | Planum temporale | -56.674 | -16.136 | 5.952 |
| e5 | Planum temporale | -56.644 | -15.429 | 6.323 |
| e6 | Transverse temporal sulcus | -54.660 | -15.243 | 7.640 |
| e7 | Anterior transverse temporal gyrus (Heschl) | -53.359 | -15.361 | 8.378 |
| e8 | Anterior transverse temporal gyrus (Heschl) | -46.154 | -13.063 | 0.720 |
| e9 | Anterior transverse temporal gyrus (Heschl) | -46.145 | -13.101 | -0.137 |
| e10 | Inferior temporal gyrus | -57.041 | -27.015 | -28.353 |
| e11 | Inferior temporal gyrus | -54.381 | -37.865 | -17.880 |
| e12 | Superior temporal gyrus | -52.614 | -46.017 | 9.537 |
| e13 | Superior temporal gyrus | -48.099 | -47.950 | 10.858 |
| e14 | Superior temporal gyrus | -38.066 | 22.806 | -16.387 |
| e15 | Subcentral gyrus (insula in the Desikan index) | -37.738 | -16.644 | -3.179 |
| e16 | Triangular part of the inferior frontal gyrus | -51.937 | 24.225 | 21.061 |
| e17 | Precentral gyrus | -28.281 | 17.303 | -25.166 |
| e18 | Straight gyrus | -3.673 | 41.258 | -27.170 |
| e19 | Lateral occipito-temporal gyrus (fusiform in the Desikan index) | -39.806 | -14.984 | -35.240 |
| e20 | (Claustrum in the Desikan index) | -37.868 | -17.628 | -2.209 |
| e21 | Collateral sulcus (parahippocampal in the Desikan index) | -16.591 | -18.308 | -25.549 |

**Table A.** Coordinates (MNI) of the intracranial electrodes for Patient 1 (left hemisphere). Electrode names correspond to the ones in Figures 3 and 4. Electrodes were labeled according to the Destrieux atlas, with some clarifications taken from the Desikan atlas where appropriate.

| Electrode | Name | x (mm) | y (mm) | z (mm) |
| --- | --- | --- | --- | --- |
| e1 | Planum temporale | 53.629 | -21.618 | 8.327 |
| e2 | Planum temporale | 51.681 | -24.265 | 9.667 |
| e3 | Planum temporale | 50.515 | -25.302 | 9.905 |
| e4 | Planum temporale | 48.230 | -27.210 | 10.570 |
| e5 | Planum temporale | 45.422 | -27.747 | 11.074 |
| e6 | Superior temporal gyrus | 48.464 | 7.276 | -14.409 |
| e7 | Central operculum (postcentral in the Desikan index) | 66.207 | -11.345 | 13.206 |
| e8 | Central operculum (postcentral in the Desikan index) | 63.616 | -12.211 | 13.705 |
| e9 | Central operculum (supramarginal gyrus in the Desikan index) | 62.072 | -12.605 | 16.220 |
| e10 | Central operculum (supramarginal gyrus in the Desikan index) | 55.452 | -20.573 | 8.039 |
| e11 | Superior segment of the circular sulcus of the insula | 39.075 | 22.383 | -0.847 |
| e12 | Opercular part of the inferior frontal gyrus | 55.930 | 17.035 | 7.396 |
| e13 | Triangular part of the inferior frontal gyrus | 52.312 | 27.562 | 1.583 |
| e14 | Triangular part of the inferior frontal gyrus | 45.710 | 26.277 | 3.959 |
| e15 | Inferior frontal sulcus | 44.085 | 22.236 | 35.566 |
| e16 | Inferior frontal sulcus | 43.651 | 27.364 | 36.272 |
| e17 | Superior frontal sulcus | 25.234 | 21.625 | 44.623 |
| e18 | Superior frontal sulcus | 23.314 | 40.623 | 35.091 |
| e19 | Orbital sulci | 13.636 | 22.641 | -26.543 |
| e20 | Straight gyrus | 7.988 | 49.162 | -23.188 |
| e21 | Superior frontal gyrus | 6.981 | 16.635 | 50.347 |
| e22 | Hippocampus | 33.043 | -20.866 | -17.369 |
| e23 | Hippocampus | 28.016 | -21.550 | -18.824 |
| e24 | Hippocampus | 23.606 | -22.151 | -20.711 |
| e25 | Hippocampus | 33.416 | -8.267 | -18.194 |

**Table B.** Coordinates (MNI) of the intracranial electrodes for Patient 2 (right hemisphere). Electrode names correspond to the ones in Figures 3 and 4. Electrodes were labeled according to the Destrieux atlas, with some clarifications taken from the Desikan atlas where appropriate.

| Electrode | Name | x (mm) | y (mm) | z (mm) |
| --- | --- | --- | --- | --- |
|  | Left hemisphere |  |  |  |
| e1 | Superior temporal gyrus | -64.873 | -14.089 | 0.231 |
| e2 | Superior temporal gyrus | -63.148 | -11.943 | -2.450 |
| e3 | Superior temporal gyrus | -60.376 | -12.702 | -5.622 |
| e4 | Superior temporal gyrus | -57.995 | -13.194 | -7.361 |
| e5 | Planum temporale | -55.966 | -17.136 | 5.509 |
| e6 | Planum temporale | -54.646 | -17.602 | 6.319 |
| e7 | Anterior transverse temporal gyrus (Heschl) | -54.101 | 26.131 | 17.053 |
| e8 | Anterior transverse temporal gyrus (Heschl) | -50.834 | 26.552 | 19.610 |
| e9 | Anterior transverse temporal gyrus (Heschl) | -46.387 | -12.549 | 0.507 |
| e10 | Anterior transverse temporal gyrus (Heschl) | -45.951 | -13.275 | 2.016 |
| e11 | Pars opercularis | -45.090 | -14.600 | 3.662 |
| e12 | Pars opercularis | -41.133 | -13.632 | 5.850 |
|  | Right hemisphere |  |  |  |
| e13 | Lateral orbitofrontal | 24.656 | 33.118 | -14.822 |
| e14 | Lateral occipito-temporal gyrus (fusiform in the Desikan index) | 37.478 | -36.916 | 39.928 |
| e15 | Lateral occipito-temporal gyrus (fusiform in the Desikan index) | 37.787 | -31.468 | 40.523 |
| e16 | Supramarginal gyrus | 42.246 | -6.612 | -6.982 |
| e17 | Supramarginal gyrus | 43.013 | 38.520 | 7.249 |
| e18 | Triangular part of the inferior frontal gyrus | 43.206 | -13.393 | 9.954 |
| e19 | Anterior transverse temporal gyrus (Heschl) | 44.441 | -46.198 | -19.325 |
| e20 | Anterior transverse temporal gyrus (Heschl) | 44.567 | -45.364 | -16.674 |
| e21 | Anterior transverse temporal gyrus (Heschl) | 50.059 | -10.834 | -0.677 |
| e22 | Anterior transverse temporal gyrus (Heschl) | 50.059 | -10.834 | -0.677 |
| e23 | Anterior transverse temporal gyrus (Heschl) | 58.247 | -13.087 | 5.508 |
| e24 | Planum temporale | 58.247 | -13.087 | 5.508 |
| e25 | Planum temporale | 59.086 | -13.018 | 5.520 |
| e26 | Planum temporale | 59.086 | -13.018 | 5.520 |
| e27 | Superior temporal gyrus | 60.594 | -20.266 | -3.667 |
| e28 | Superior temporal gyrus | 60.707 | -19.479 | -3.578 |
| e29 | Superior temporal gyrus | 60.707 | -19.479 | -3.578 |
| e30 | Superior temporal gyrus | 62.414 | -20.083 | -3.436 |
| e31 | Superior temporal gyrus | 63.058 | -20.326 | -3.229 |
| e32 | Superior temporal gyrus | 64.337 | -20.501 | -4.927 |
| e33 | Superior temporal gyrus | 68.547 | -20.830 | -2.812 |

**Table C.** Coordinates (MNI) of the intracranial electrodes for Patient 3. Electrode names correspond to the ones in Supplementary Figure 2. Electrodes were labeled according to the Destrieux atlas, with some clarifications taken from the Desikan atlas where appropriate.
